# A decade long insight into patient views on kidney cancer care delivery

**DOI:** 10.1111/bju.16530

**Published:** 2024-09-12

**Authors:** Sabrina H. Rossi, Geraldine Fox, Malcolm Packer, Andrew Greaves, Maxine Tran, Natalie Charnley, Grenville Oades, Ekaterini Boleti, Grant D. Stewart

**Affiliations:** ^1^ Department of Surgery University of Cambridge Cambridge UK; ^2^ CRUK Cambridge Centre University of Cambridge Cambridge UK; ^3^ Kidney Cancer UK Guildford UK; ^4^ UCL Division of Surgery and Interventional Science, Royal Free Hospital University College London London UK; ^5^ Department of Urology Lancashire Teaching Hospitals NHS Foundation Trust Preston UK; ^6^ Queen Elizabeth University Hospital Glasgow UK; ^7^ Royal Free Hospital London UK

**Keywords:** kidney cancer, patient and public involvement, early diagnosis, equitable care, public engagement

**Abbreviations**
PPIE: public involvement and engagement
SACT: systemic anti‐cancer therapy

Patient and public involvement and engagement (PPIE) is crucial to ensure that patient care and research is relevant to patient needs and is more likely to have a positive impact. Indeed, previous research priority setting initiatives in kidney cancer, which have led to tangible research programmes, have actively involved patients and their carers [1]. A continued focus on the patients and the public perspective of kidney cancer diagnosis and treatment is imperative. Kidney Cancer UK, a UK kidney cancer charity, carried out a survey of patients with kidney cancer annually over the last decade. Here, we report longitudinal survey results and reflect on the future direction of kidney cancer care and how they link to research priorities in the UK.

Kidney Cancer UK delivered an annual patient survey, distributed on‐line via the QuestionPro platform and via post, between 2014 and 2023. This was publicised via social media platforms (including the charity's website, Facebook, Instagram and X). Additionally, the survey was sent to clinicians and cancer nurse specialists for distribution to patients. The questionnaire focused on the patients’ experience on diagnosis, treatment and information/support received. Survey participation increased over time, with 68 completed in 2013, >300 participants from 2019 onwards and >500 participants in 2022 and 2023.

A consistent survey finding was that patients feel the pathway to diagnosis for kidney cancer could be improved. Interestingly, the proportion of respondents waiting >3 months for a specialist kidney cancer diagnosis underwent a sharp rise in 2020, increasing from around 11–18% in the preceding years to 33%, with levels remaining stable since then (Fig. 1). This may be partly related to delays in diagnosis following the coronavirus pandemic, however, there was no improvement in subsequent years, perhaps reflecting growing NHS pressures. NHS data suggest an increased backlog with growing waiting times to see a specialist in secondary care and increased waiting lists for treatment [2]. Timely diagnosis remains a key area for improvement and is a target for NHS England. One in four patients reported that their GP initially misdiagnosed their symptoms as an alternative condition, and they felt that this delayed their diagnosis. This proportion has remained static over the last 4 years (Fig. 1). Indeed, kidney cancer is often asymptomatic or results in non‐specific symptoms. Only 19% of survey participants reported haematuria. This is in keeping with UK data demonstrating that only 23% of patients diagnosed with renal cancer report visible haematuria, and this is more commonly associated with advanced tumour stage (49% having Stage III–IV disease) [3]. Although increased public awareness of kidney cancer symptoms has been successfully achieved through public health ‘Blood in the Pee’ Campaigns in England, this was associated with increased early‐stage diagnoses for bladder but not kidney cancer [4]. Different strategies have been proposed aiming to increase early detection, including the development of risk prediction models to aid GPs to predict the risk of kidney (and bladder) cancer based on clinical signs and symptoms in primary care, to aid diagnostic patient triage. Several such models with good discrimination (area under the receiver operating curve >0.8) have been identified [5]. For example, a model by Hippisley‐Cox et al. [5] included demographic and lifestyle risk factors with clinical features such as smoking, haematuria, and abdominal pain; however, further external validation is necessary prior to adoption of any models in clinical practice. Given most kidney cancer cases are diagnosed incidentally [3], the Kidney Cancer UK patient survey emphasised the need for research focusing on earlier kidney cancer detection and improved patient pathways to diagnosis. This is in keeping with results of a priority setting initiative published in 2020, which identified earlier cancer diagnosis as a key research priority [1].

**Fig. 1:**
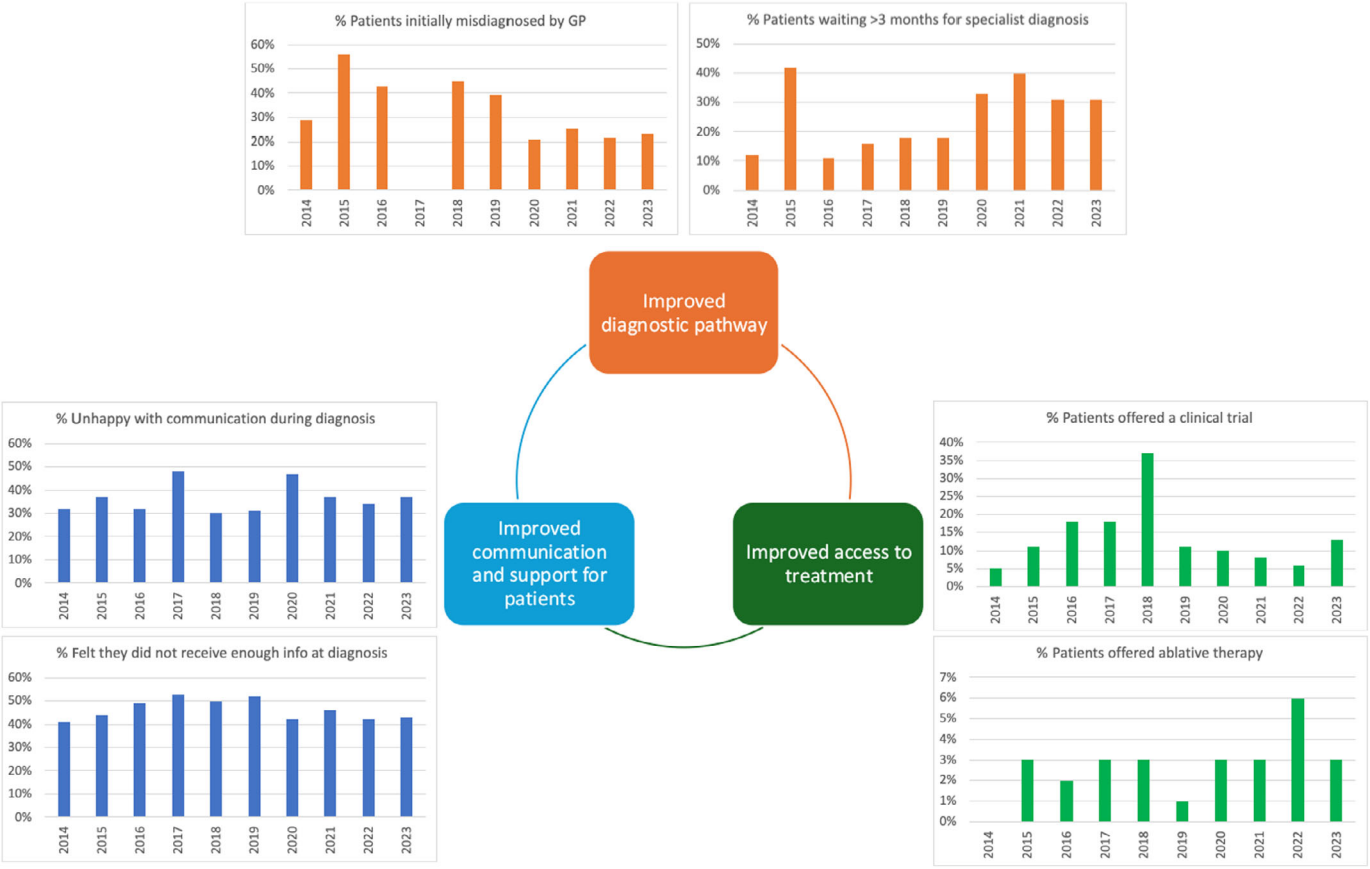
Summary of patient survey results and main suggestions for kidney cancer research and patient care.

The survey highlighted increased availability of certain treatment options for kidney cancer, including increased use of robotic surgery and systemic anti‐cancer therapy (SACT), over the 10‐year study period. 25% of patients reported receiving robotic surgery in 2023, compared to 4% in 2016. Similarly, SACT use increased from 8% in 2014, to 41% in 2018 and remained approximately static since then. However, great regional variation in access to SACT has been demonstrated across the UK, with rates of SACT in patients with metastases varying between 40% and 71% [6]. More equitable access to SACT is a priority highlighted by both patients and healthcare professionals alike.

The survey also suggested that access to clinical trials has remained very low throughout the last decade. Whilst 5% of participants stated they were offered access to a clinical trial in 2014, this percentage remained largely static over the last decade, with 13% offered access to a clinical trial in 2023 (Fig. 1). This is a key area highlighted for improvement. Furthermore, the use of ablative therapies did not increase, with <6% of patients reporting having received ablative therapy in the 10‐year period (Fig. 1). These data are consistent with results from the Kidney Cancer UK Quality Performance Audit of kidney cancer services in England, in which only 3% of T1 tumours were treated with ablative therapy [6]. Indeed improved characterisation, diagnosis and management of small renal masses has been previously identified as a research priority [1].

The final salient point from a decade of this survey was the need for improved communication and support for patients. In 2023, 37% of patients reported they were unhappy with the way their diagnosis was communicated. This proportion remained largely static across the last decade (Fig. 1). Similarly, 41–53% of patients felt they were not given enough information at diagnosis, and these rates also remained static over the last decade. For example, in 2023, 43% of participants reported not being given information leaflets at the time of diagnosis (Fig. 1). A general decline was noted in the proportion of patients who were given details regarding a named key worker or cancer nurse specialist from >40% in 2014 to 21% in 2023. Provision of patient information leaflets, access to a named key worker and good communication are all key audit targets in kidney cancer care in the UK. Independent patient surveys also echoed poor communication in the NHS [7] and it is postulated this may be secondary to growing pressures on the healthcare system. A study of complaints in the NHS demonstrated that nearly half of these (48%) are related to poor communication [8]. These survey results are therefore crucial in highlighting the need for better communication and provision of information and support for patients with kidney cancer.

In summary, these longitudinal survey data highlight the need for improvements in the early detection and diagnostic pathway for patients with kidney cancer, better patient communication and support, and more equitable access to treatment options (Fig. 1). These data are not routinely captured by audits and clinical research, highlighting the importance of PPIE. We acknowledge the survey has some limitations, namely that respondents were a self‐selected group of patients and demographics (including stage at diagnosis) were unknown. Despite this, access to 10‐year longitudinal data highlights trends over time, including the potential impact of the pandemic on patient care and represents a strength of this work.

## Disclosure of Interests

Grant D. Stewart has received educational grants from Pfizer, AstraZeneca, and Intuitive; consultancy fees from Pfizer, Merck, EUSA Pharma, and CMR Surgical; travel expenses from Pfizer; and speaker fees from Pfizer. Natalie Charnley has received grants from Ipsen, MSD and BMS. Malcolm Packer and Andrew Greaves are employed by the Kidney Cancer UK charity, and Maxine Tran, Natalie Charnley, Grenville Oades, Geraldine Fox and Ekaterini Boleti are charity trustees. All other co‐authors have no relevant conflicts of interest.
